# A microRNA panel compared to environmental and polygenic scores for colorectal cancer risk prediction

**DOI:** 10.1038/s41467-021-25067-8

**Published:** 2021-08-10

**Authors:** Janhavi R. Raut, Ben Schöttker, Bernd Holleczek, Feng Guo, Megha Bhardwaj, Kaya Miah, Petra Schrotz-King, Hermann Brenner

**Affiliations:** 1 Division of Preventive Oncology, German Cancer Research Center (DKFZ) and National Center for Tumor Diseases (NCT), Heidelberg, Germany; 2 Medical Faculty Heidelberg, University of Heidelberg, Heidelberg, Germany; 3 Division of Clinical Epidemiology and Aging Research, German Cancer Research Center (DKFZ), Heidelberg, Germany; 4 Network Aging Research, University of Heidelberg, Heidelberg, Germany; 5 Saarland Cancer Registry, Saarbrücken, Germany; 6 Division of Biostatistics, German Cancer Research Center (DKFZ), Heidelberg, Germany; 7 German Cancer Consortium (DKTK), German Cancer Research Center (DKFZ), Heidelberg, Germany

**Keywords:** Biomarkers, Cancer epidemiology, Cancer prevention

## Abstract

Circulating microRNAs (miRNAs) could improve colorectal cancer (CRC) risk prediction. Here, we derive a blood-based miRNA panel and evaluate its ability to predict CRC occurrence in a population-based cohort of adults aged 50–75 years. Forty-one miRNAs are preselected from independent studies and measured by quantitative-real-time-polymerase-chain-reaction in serum collected at baseline of 198 participants who develop CRC during 14 years of follow-up and 178 randomly selected controls. A 7-miRNA score is derived by logistic regression. Its predictive ability, quantified by the optimism-corrected area-under-the-receiver-operating-characteristic-curve (AUC) using .632+ bootstrap is 0.794. Predictive ability is compared to that of an environmental risk score (ERS) based on known risk factors and a polygenic risk score (PRS) based on 140 previously identified single-nucleotide-polymorphisms. In participants with all scores available, optimism-corrected-AUC is 0.802 for the 7-miRNA score, while AUC (95% CI) is 0.557 (0.498–0.616) for the ERS and 0.622 (0.564–0.681) for the PRS.

## Abstract (web summary)

Appropriate risk models could facilitate risk stratification for colorectal cancer (CRC) screening. Here, the authors propose a blood-based microRNA signature observed to have altered expression in pre-diagnostic samples, which might be useful to identify high-risk populations for colorectal cancer screening.

## Introduction

Colorectal cancer (CRC) is the third most common incident cancer and the second leading cause of cancer mortality worldwide, accounting for 1.85 million incident cases and ~880,000 deaths in 2018^1^. The disease burden can be decreased with population-based screening, which has been demonstrated to be effective in reducing mortality and potentially preventing the occurrence of CRC^2–4^. Currently, colonoscopy is regarded as the gold standard method for early diagnosis of CRC, but its widespread use is limited by its invasive nature, dietary restriction requirement, and costs^5–7^. While fecal immunochemical test for hemoglobin has been proven to be an effective, currently available non-invasive test to screen patients who are at average risk for the development of CRC, it has limited sensitivity to detect advanced adenomas or stage I CRCs^8,9^. In order to maximize screening benefits and minimize harms and costs, alternative minimally invasive or non-invasive tests that can more accurately define low- and high-risk populations are needed.

Risk models based on genetic susceptibility loci, alone or in combination with environmental risk factors have been increasingly propagated for risk stratification in CRC screening. However, the models used so far have generally yielded limited ability to distinguish between individuals with and without CRC and its precursors^10–13^. In recent years, blood levels of microRNAs (miRNAs) have been linked to CRC development^14,15^. and have consistently shown some potential at distinguishing CRC patients and controls free of colorectal neoplasms^16–21^. However, most previous studies have examined circulating miRNA levels in patients with an established CRC diagnosis, making it impossible to determine if they will be useful for risk stratification or are a result of cancer progression. Recently, Wikberg et al.^22^ showed that plasma levels of miRNAs were not only different in CRC patients at the moment of diagnosis but, also, they were altered several years before diagnosis. However, major changes in miRNA levels between samples collected years before diagnosis and samples collected at the time of diagnosis were observed among the majority of cases and seemed to occur mainly in the three years prior to diagnosis. Also, differences in miRNA levels between pre-diagnostic plasma samples and control plasma samples were generally quite different from differences between diagnostic plasma samples and control plasma samples. It is therefore unclear, if and to what extent, blood-based miRNA signatures might enable CRC risk prediction years before diagnosis.

In this study, we derive and validate a blood-based microRNA signature predicting CRC occurrence over up to 14 years of follow-up in a large population-based cohort study of older adults. In addition, we compare its predictive performance with that of a recently developed polygenic risk score (PRS) and an established environmental risk score (ERS).

## Results

### Characteristics of study populations

The characteristics of populations from the discovery and prospective sets are shown in Table 1. The discovery set included 20 newly diagnosed CRC cases (from the GEKKO (Gebt dem Krebs keine Chance—Onkocheck) study arm B) and 20 controls free of colorectal neoplasms (from the GEKKO study arm A) matched by age and sex. Of the 19 cases with information about tumor stage at diagnosis, one was classified as stage 0, one as stage I, nine as stage II, four as stage III, and four as stage IV. The prospective set included 198 participants with incident CRC and 178 randomly selected participants without diagnosis of CRC identified within 14 years of follow-up in the ESTHER (Epidemiologische Studie zu Chancen der Verhütung, Früherkennung und optimierter Therapie chronischer Erkrankungen in der älteren Bevölkerung) study. By the 8-year follow-up, 62 cases (31.3%) and 95 controls (53.4%) had reported to have ever undergone a screening colonoscopy. For the incident cases, the time between sample collection and diagnosis ranged from 0.0 to 14.3 years (median (interquartile range), 6.8 (3.3–9.6) years). Of the 153 cases with information about tumor stage at diagnosis, 14 were classified as stage 0, 20 as stage I, 61 as stage II, 30 as stage III, and 28 as stage IV. Information on 140 relevant single-nucleotide polymorphisms (SNPs) used to build PRS was not available for 21 participants (cases *n* = 17; controls, *n* = 4). Therefore, the study population for the analyses on all scores (*n* = 355, cases *n* = 181; controls, *n* = 174) was smaller than the overall prospective set population. The distribution of characteristics was largely similar across both, the discovery and prospective sets with the mean age at sampling being around 65 years and males representing >50% of population in both sets.

**Table 1:** Characteristics of the study populations.

Characteristics	Discovery set		Characteristics	Prospective set	
	CRC Cases (*n* = 20)	Controls (*n* = 20)		CRC Cases (*n* = 198)	Controls (*n* = 178)
Age at sampling			Age at sampling		
			Mean (SD)	64.6 (5.9)	62.2 (6.6)
Mean (SD)	64.8 (12.3)	64.7 (12.1)	Median (range)	65 (50–75)	62 (50–75)
			Age at diagnosis		
Median (range)	64 (47–88)	64 (47–87)	Mean (SD)	71.3 (6.8)	–
			Median (range)	71.3 (53–86)	–
Gender– counts (%)			Gender– counts (%)		
Male	11 (55.0)	11 (55.0)	Male	122 (61.6)	89 (50.0)
Female	9 (45.0)	9 (45.0)	Female	76 (38.4)	89 (50.0)
TNM stage– counts (%)			TNM stage at diagnosis– counts (%)		
Stage 0	1 (5.0)	–	Stage 0	14 (7.1)	–
Stage I	1 (5.0)	–	Stage I	20 (10.1)	–
Stage II	9 (45.0)	–	Stage II	61 (30.8)	–
Stage III	4 (20.0)	–	Stage III	30 (15.2)	–
Stage IV	4 (20.0)	–	Stage IV	28 (14.1)	–
Unknown	1 (5.0)	–	Unknown	45 (22.7)	–

*CRC* colorectal cancer, *n* number, *SD* standard deviation, *TNM* Tumor Nodes Metastasis classification.

### Selection of miRNA candidates

In the discovery phase, we identified and selected 20 miRNAs differentially expressed from next-generation sequencing (NGS) profiling of discovery set samples (Supplementary Table 1) and 21 miRNAs reported to be differentially expressed in the literature (Supplementary Table 2) for quantitative real-time polymerase chain reaction (qPCR) profiling in the prospective set.

### qPCR quality controls

**Fig. 1:**
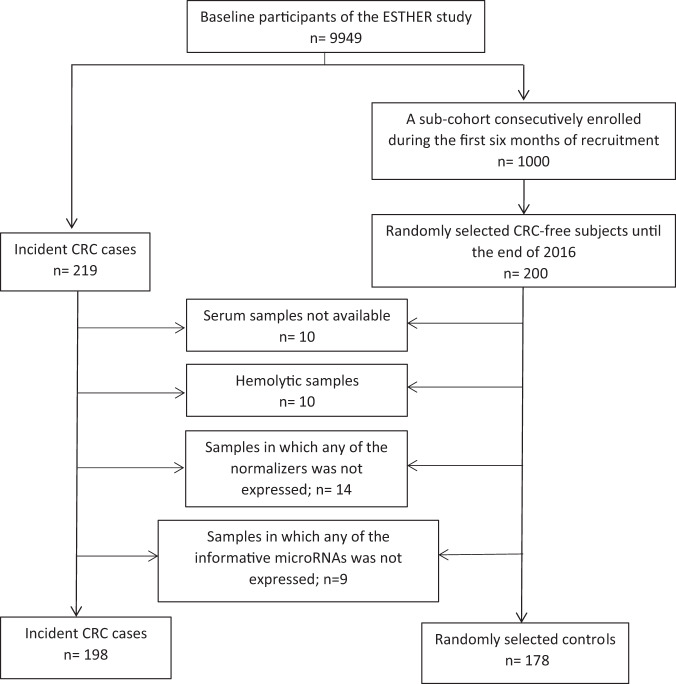
Flow diagram for selection of prospective set participants. *CRC* colorectal cancer.

RNA extraction efficiency, monitored using UniSp2 and UniSp4, was acceptable with raw quantification cycle (Cq) values being consistent across the dataset (UniSp2: Cq 21.26 ± 1.93, UniSp4: Cq 28.42 ± 2.82). UniSp6 was used to monitor the complementary DNA (cDNA) synthesis reactions and indicated constant efficiency of the reverse transcription step with no signs of inhibition (Cq 18.15 ± 0.11). Ten samples displayed significant hemolysis (mean Cq_miR‑23a_ − mean Cq_miR‑451a_ > 7) and were excluded from downstream analysis (Fig. 1).

### Development of the microRNA risk score (miR-score) in the prospective set

Of the 41 miRNAs evaluated, three (miR-93-5p, miR-1246, and miR-223-3p) were selected as normalizers. Of the remaining 38 candidate miRNAs, seven were detectable (Cq value < 40) in at least 99% of the samples and were identified as informative miRNAs. Samples with missing values for any of the informative miRNAs were excluded from further analysis (*n* = 9) and the remaining were included in the prospective set. The data were normalized to the average Cq value of the normalizers. The informative miRNAs were introduced as a panel into a logistic regression model on CRC risk, based on the prospective set. Using the observed weights from the regression model, a miR-score was calculated for each participant (linear predictor):

miR-score = 0.1899 + let-7g-5p*0.2351 + miR-19a-3p*-0.2024 + miR-23a-3p*1.6595 + miR-92a-3p*0.4794 + miR-144-5p*0.2002 + miR-21-5p*-1.6772 + miR-27a-3p*0.1014

### Associations of the risk scores with CRC incidence

The associations of the risk scores with CRC incidence in the subpopulation including prospective set participants with all scores available are presented in Table 2. The middle quintiles (Q3) were assigned as the reference group in each set. For model 1, having a miR-score in the fifth quintile (Q5) was associated with a significantly increased risk of CRC [odds ratio (OR), 7.20 (95% confidence interval (CI), 3.60–14.39)]. Additionally, having a miR-score in the first quintile (Q1) was associated with a significantly decreased risk of CRC [OR, 0.33 (95% CI, 0.12–0.95)]. For model 2, with additional adjusting for age and sex, the miR-score remained a strong and significant predictor with an OR (95% CI) of 7.20 (3.56–14.59). Associations for the ERS were much weaker and did not reach statistical significance. However, for model 1, having a PRS in the first quintile (Q1) was associated with a significantly decreased risk of CRC [OR, 0.46 (95% CI, 0.21–0.97)]. Nevertheless, with additional adjusting for age and sex in model 2, the association did not remain significant.

**Table 2:** Individual associations of miR-score, ERS and PRS with CRC incidence in prospective set participants with all scores available (*N*_cases/controls_ = 181/174).

Population	Quintile^a^	Cases	Controls	Model 1^b^		Model 2^c^	
				OR (95% CI)^d^	*P* value^d^	OR (95% CI)^d^	*P* value^d^
miR-score	Q1 (< −1.38]	6 (3.3)	36 (20.7)	0.33 (0.12–0.95)	0.0388	0.34 (0.12–0.97)	0.0439
	Q2 (−1.38, −0.91]	14 (7.7)	34 (19.5)	0.82 (0.35–1.93)	0.6552	0.88 (0.37–2.10)	0.7759
	Q3 (−0.91, −0.41]	17 (9.4)	34 (19.5)	Ref.		Ref.	
	Q4 (−0.41, 0.06]	18 (9.9)	35 (20.1)	1.03 (0.46–2.32)	0.9459	1.09 (0.48–2.50)	0.8352
	Q5 (> 0.06)	126 (69.6)	35 (20.1)	7.20 (3.60–14.39)	2.28E-08	7.20 (3.56–14.59)	4.19E-08
ERS	Q1 (<3]	34 (18.8)	42 (24.1)	0.83 (0.44–1.59)	0.580	–	–
	Q2 (3, 4]	30 (16.6)	34 (19.5)	0.91 (0.46–1.78)	0.779	–	–
	Q3 (4, 5]	35 (19.3)	36 (20.7)	Ref.		–	–
	Q4 (5, 7]	46 (25.4)	34 (19.5)	1.39 (0.73–2.65)	0.314	–	–
	Q5 (>7)	36 (19.9)	28 (16.1)	1.32 (0.67–2.61)	0.419	–	–
PRS	Q1 (<7.66]	16 (8.8)	35 (20.1)	0.46 (0.21–0.97)	0.042	0.49 (0.22–1.05)	0.0680
	Q2 (7.66, 7.92]	24 (13.3)	34 (19.5)	0.71 (0.35–1.42)	0.331	0.76 (0.37–1.56)	0.4543
	Q3 (7.92, 8.15]	35 (19.3)	35 (20.1)	Ref.		Ref.	
	Q4 (8.15, 8.46]	47 (26.0)	35 (20.1)	1.34 (0.71–2.55)	0.367	1.24 (0.64–2.40)	0.5250
	Q5 (>8.46)	59 (32.6)	35 (20.1)	1.69 (0.90–3.16)	0.103	1.75 (0.92–3.34)	0.0880

*miR-score* microRNA risk score, *ERS* environmental risk score, *PRS* polygenic risk score, *CRC* colorectal cancer, *OR* odds ratio, *CI* confidence interval, *Q* quintile, *Ref.* reference category.

^a^Quintiles of risk score among controls.

^b^Model 1: without adjustment for any confounders.

^c^Model 2: like model 1, adjusted for age and sex.

^d^OR, 95% CI and two-sided *P* values were generated from logistic regression model.

### Risk prediction by individual and combined risk scores for CRC

**Fig. 2:**
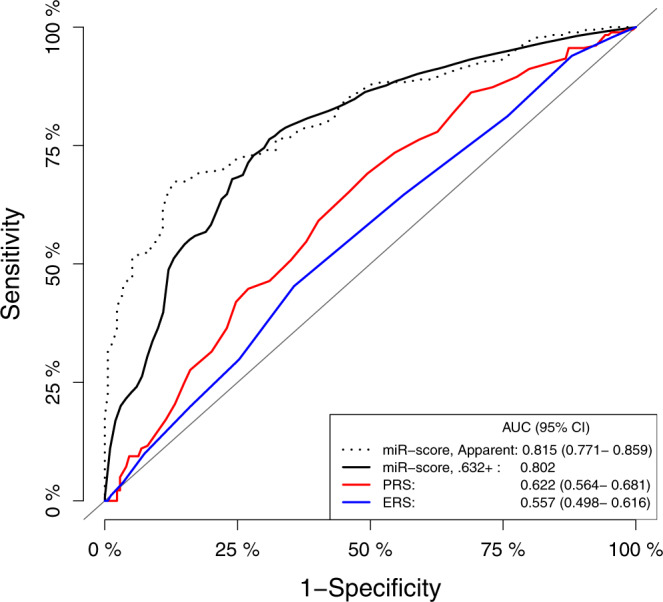
Performance of risk scores for predicting CRC risk. Receiver operating characteristic curves for CRC risk prediction in prospective set participants with all scores available (*N*_cases/controls_ = 181/174) according to microRNA risk score (miR-score), polygenic risk score (PRS) and environmental risk score (ERS).

The predictive performances of individual risk scores and score combinations are presented in Table 3. In the prospective set, the miR-score showed a high predictive performance with an optimism-corrected area under the receiver-operating-characteristic curve (AUC) of 0.794 and a Brier score of 0.184. Additionally, consistent performance was observed in specific sub-groups defined by follow-up time restricted to the initial three years after recruitment and to subsequent years (Supplementary Table 3). In the subpopulation including prospective set participants with all scores available, AUC was the lowest for ERS alone and the PRS performed slightly better than ERS (Fig. 2). Combining PRS with ERS improved the predictive performance to a very limited extent [AUC_ERS + PRS_ = 0.631 (95% CI, 0.573–0.689) vs. AUC_ERS_ = 0.557 (95% CI, 0.498–0.616) and AUC_PRS_ = 0.622 (95% CI, 0.564–0.681)]. The miR-score [optimism-corrected AUC = 0.802] substantially outperformed ERS, PRS, and their combination. Compared to the model based on miR-score alone, models combining miR-score with ERS, or PRS, or both yielded optimism-corrected AUCs of 0.814, 0.822, and 0.820, respectively, and resulted only in a minimal increase in performance.

**Table 3:** Risk prediction by individual and combined risk scores for CRC.

Population	Predictor	AUC (95% CI)	Brier score
Prospective set (*N*_cases/controls_ = 198/178)	miR-score	Apparent: 0.808 (0.765–0.851); 0.632+: 0.794	Apparent: 0.175; 0.632+: 0.184
Prospective set participants with all scores available (*N*_cases/controls_ = 181/174)	ERS	0.557 (0.498–0.616)	0.248
	PRS	0.622 (0.564–0.681)	0.240
	ERS + PRS	0.631 (0.573–0.689)	0.238
	miR-score	Apparent: 0.815 (0.771–0.859); 0.632+: 0.802	Apparent: 0.172; 0.632+: 0.181
	ERS + miR-score	Apparent: 0.815 (0.771–0.859); 0.632+: 0.814	Apparent: 0.172; 0.632+: 0.174
	PRS + miR-score	Apparent: 0.824 (0.782–0.867); 0.632+: 0.822	Apparent: 0.169; 0.632+: 0.171
	ERS + PRS + miR-score	Apparent: 0.824 (0.781–0.867); 0.632+: 0.820	Apparent: 0.169; 0.632+: 0.172

*miR-score* microRNA risk score, *ERS* environmental risk score, *PRS* polygenic risk score, *CRC* colorectal cancer, *AUC* area under the receiver-operating-characteristic curve, *CI* confidence interval.

Note– miR-score was derived in the overall prospective set (N_cases/controls_ = 198/178).

### Deregulations of individual informative miRNAs in the prospective set: comparison with discovery set and literature results

In the prospective set, evaluation of fold changes and corresponding *P* values of each individual informative miRNA (Table 4) revealed that upregulation of three miRNAs (let-7g-5p, miR-23a-3p, and miR-92a-3p) in CRC cases versus controls was consistent with findings from previous studies^23–29^. Upregulation of miR-144-5p was consistent with our discovery set results. However, we observed downregulation of miR-19a-3p and miR-21-5p in CRC cases versus controls, which have been previously reported to be upregulated in other studies^17,22,28–34^. Furthermore, we observed downregulation of miR-27a-3p, previously reported to be upregulated in a study by Vychytilova-Faltejskova et al.^27^, but downregulated in a study by Tan et al.^35^. The miRNA expression levels in serum of cases and controls from the prospective set (normalized to the average Cq value of miR-93-5p, miR-1246, and miR-223-3p) are shown in Supplementary Fig. 1. In the prospective set, expression levels of some informative miRNAs showed significant correlations with each other as assessed by the pairwise calculation of Spearman’s rank correlation coefficients (Supplementary Fig. 2). High significant positive correlation (r_s_ = 0.73) was observed between miR-21-5p and miR-27a-3p.

**Table 4:** Deregulation of each individual miRNA from the miR-score and comparison with results in the literature.

	Candidate type	Deregulation in the literature/ Discovery set	Results in the Prospective set (*N*_cases/controls_ = 198/178)			
			Deregulation	Fold change	*P* value^a^	Corrected *P* value^b^
let-7g-5p	Literature	↑^28^	↑	1.26	5.04E-06	2.02E-05
miR-19a-3p	Literature	↑^17,29,30,34^	↓	0.61	2.01E-14	1.41E-13
miR-23a-3p	Literature	↑^25,27^	↑	1.21	1.26E-05	3.77E-05
miR-92a-3p	Literature	↑^23,24,26,29^, ↓^28^	↑	1.11	2.72E-02	2.72E-02
miR-144-5p	NGS	↑	↑	1.53	7.88E-08	3.94E-07
miR-21-5p	Literature	↑^22,28,31–34,43^	↓	0.69	2.94E-10	1.76E-09
miR-27a-3p	Literature	↑^27^, ↓^35^	↓	0.82	3.74E-05	7.48E-05

↑represents significant upregulation. ↓represents significant downregulation.

^a^Values were generated from two-sided Mann–Whitney test.

^b^Multiple testing correction by the method of Bonferroni–Holm^64^.

## Discussion

In this study, we report the potential of circulating miRNAs in predicting CRC occurrence by analyzing candidate miRNAs in samples collected several years before a CRC diagnosis. In our two-step investigation of miRNA expression, we first selected 20 differentially expressed miRNAs from NGS profiling of discovery set (retrospective samples) and 21 miRNAs reported to be differentially expressed in the literature for qPCR profiling. In the second step, we evaluated the selected miRNAs in serum samples of 385 participants drawn from a prospective cohort with 14-year follow up. Consideration of a miR-score incorporating seven miRNAs expressed in 99% of included samples (let-7g-5p, miR-19a-3p, miR-23a-3p, miR-92a-3p, miR-144-5p, miR-21-5p and miR-27a-3p) yielded an optimism-corrected AUC of 0.794 for CRC risk prediction in the prospective set. Next, we compared predictive ability of the miR-score with predictive ability of the modified version of a previously derived ERS for CRC^36^ and a PRS based on 140 independent SNPs previously reported in association to CRC^37^. In our prospective investigation, we found strong associations of the miR-score with the risk of CRC also after adjustment for age and sex. Furthermore, this study demonstrated that the miR-score was highly predictive for CRC risk and strongly enhanced the risk prediction compared to the risk stratification by ERS, PRS, and their combination.

The method used to detect and analyze miRNA expression levels strongly influences the outcome of the studies. Compared to microarrays and qPCR where targets are pre-selected, the use of high-throughput NGS enables both discovering novel miRNAs and acquiring a quantitative estimate of known miRNA species in an unbiased manner^38^. In our study, genome-wide plasma miRNA profiling was performed with Illumina NextSeq 500 on the discovery set and evaluation of selected candidates in serum samples from an independent prospective set was performed with qPCR. Among the twenty prominent miRNAs that were deregulated in the discovery set, only one miRNA (miR-144-5p) was included in the miR-score developed with the prospective set. MiR-144-5p has so far been explored as a fecal-based marker^39,40^ for CRC screening. Our NGS study discovered it was upregulated in plasma of CRC cases compared to controls (Supplementary Table 1). Remarkably, the observed upregulation of miR-144-5p in the discovery set was replicated by qPCR profiling in the prospective set. Since it was upregulated not only in samples collected at the time of diagnosis, but also in pre-diagnostic samples collected in median 6.8 years prior to diagnosis, it may represent a novel blood-based marker for CRC screening. Thus, though only one miRNA from the first phase of the study was included in the established panel, it was found to be an important component that has not been previously suggested as a blood-based screening marker. Only one out of twenty NGS candidates from the discovery set being included in the established miR-score could be due to several reasons. Although both platforms are highly capable for miRNA profiling, NGS shows a lower accuracy for miRNA differential expression analysis compared to qPCR^38^. Differences in reproducibility between platforms could also be attributed to use of different fractions of blood (plasma vs. serum) and timing of sample collection in relation to diagnosis. Plasma and serum are likely to exhibit substantial differences in their miRNA content^41,42^, which could have influenced the results. Furthermore, herein, the discovery set included samples collected at the time of diagnosis, whereas the prospective set used pre-diagnostic samples collected in median 6.8 years prior to diagnosis, complicating the comparison of results.

Among the six literature candidates included in the miR-score, miR-19a-3p was also found to be differentially expressed between cases and controls of the discovery set at a False Discovery Rate (FDR) < 0.05 based on our NGS results (Supplementary Table 1). However, for the next phase of the study, we selected candidates that met the criteria of having an average trimmed mean of M (log expression ratio) value (TMM) > 10 in either case or control group and an absolute value of log_2_fold-change (|log_2_FC|) > 1. The cut-off of TMM >10 was used since counts lower than 10 in both case and control groups might be difficult to validate in a qPCR experiment, while the cut-off of |log_2_FC| > 1 was used since smaller fold changes tend to be more affected by technical variance, and hence may be at greater risk of false-positive signals. With a |log_2_FC| of 0.82, miR-19a-3p did not meet the second criterion and hence was not selected as an NGS candidate. When selecting additional candidates from the literature, we found miR-19a-3p to be part of a promising 4-miRNA panel with its diagnostic performance [AUC = 0.95 (95% CI, 0.91–0.98)] validated using an independent cohort^29^. It was also a part of other promising panels that were also validated using independent cohorts^30,34^. Although it did not show the best discriminative capacity in our discovery set with a limited sample size, it was still a promising candidate based on the literature, and hence selected as a literature candidate. Previously, it has been reported to be over-expressed in plasma/ serum of CRC cases compared to controls^17,29,30,34^. Consistent with the literature, our discovery set results, also based on case-control comparisons, revealed upregulation of miR-19a-3p in plasma from CRC cases compared to controls. Conversely, our prospective set results revealed downregulation of miR-19a-3p in serum from CRC cases compared to controls. Another literature candidate, miR-21-5p was also observed to have contradictory deregulation in the prospective set compared to previous reports. MiR-21-5p features prominently in existing literature on miRNAs in CRC and has been reported to be over-expressed in plasma/ serum of CRC^22,28,31–34,43^, as well as advanced adenoma patients^32^ compared to controls. Conversely, results from our prospective set demonstrated downregulation of miR-21-5p in serum from CRC cases compared to controls. A potential explanation for these apparently contradictory findings may be that most prior studies and our discovery set findings were based on case-control comparisons in which blood samples were taken after diagnosis whereas our prospective set findings were based on samples collected in median 6.8 years prior to diagnosis in a prospective cohort study. Recently, Wikberg et al.^22^ observed that major changes of the miRNA pattern may occur mainly in the three years prior to CRC diagnosis. Their results showed a distinct temporal pattern of increase in plasma levels of miR-21-5p during the three years prior to clinical diagnosis indicating that its levels continue to alter as the disease progresses. Moreover, our discovery and prospective set experiments used different fractions of blood (plasma vs. serum), which could have influenced the results obtained.

Concerning other literature candidates, let-7g-5p and miR-23a-3p have previously been reported to be upregulated in CRC cases versus controls^25,27,28^. In our study, both miRNAs displayed elevated levels in pre-diagnostic samples from CRC cases compared to controls, which is in line with these previous findings. MiR-92a-3p which has been previously reported to be upregulated^23,24,26,29^, or downregulated^28^ in CRC-derived serum samples compared to controls, displayed an elevation in pre-diagnostic samples in our analysis. Finally, miR-27a-3p which was previously reported to be significantly upregulated^27^ in serum, but downregulated^35^ in plasma samples from CRC cases was revealed to be downregulated in pre-diagnostic samples from CRC cases compared to controls in our study. Together, these findings suggest that miRNA alterations in our study reflect risk rather than presence of CRC and hence may differ compared to alterations in existing CRC. There is evidence from longitudinal analyses^44–47^ that some miRNAs only increase or decrease in the circulation a relatively short time prior to clinical presentation of cancer. Prior efforts to identify circulating miRNA biomarkers related to CRC detection have employed a cross-sectional design, comparing CRC-free subjects to affected individuals with blood samples collected at or after diagnosis. Since presence of advanced disease is likely to have an impact on abundance of circulating miRNAs, this approach is less useful for discovering changes related to early CRC progression. With miRNA profiles being reported to change during disease formation and propagation^48^, it is comprehensible that the expression of these miRNAs in pre-diagnostic samples is different compared to samples from established CRC cases. Suggestions of changing miRNA patterns in the years prior to CRC diagnosis are consistent with previous observations^22^ on major changes in miRNA levels between samples taken years before diagnosis and samples taken at the time of diagnosis. Other reasons that can be given to explain the discrepancies observed between literature, discovery, and prospective set findings are the differences in sample collection, handling and processing, nucleic acid extraction, quality control, detection assays, and/or analytical methods.

In recent years, PRSs, alone or in combination with ERSs, are increasingly propagated for risk stratification in CRC screening. However, the scores used so far have generally yielded limited ability to distinguish between individuals with and without CRC and its precursors^10–13^. Jeon et al.^11^ developed a model including family history, 19 lifestyle and environmental factors, and 63 CRC-associated SNPs identified in genome-wide association studies, which predicted CRC risk with an AUC of 0.63 for men and 0.62 for women. Peng et al.^49^ evaluated and directly compared the performance of published risk prediction models for advanced colorectal neoplasms in two cohorts of subjects undergoing screening colonoscopy. The AUCs ranged from 0.57 to 0.65 for all risk scores. In line with previous reports, our estimated AUCs for ERS, PRS, and their combination in relation to CRC prediction, ranged from 0.557 to 0.631. Compared to ERS, PRS, and their combination, the proposed miR-score predicted CRC risk with substantially higher accuracy with an optimism-corrected AUC of 0.802. According to these values, we present a high-performance risk model with outstanding potential for risk stratification which may be useful for risk-adapted CRC screening strategies. For example, employing our risk prediction model may help to identify populations with very high risk for whom colonoscopy (rather than less invasive tests) could be recommended as primary screening test. Other potential uses to be evaluated in further research may include definition of risk-adapted starting ages of CRC screening.

A major strength of our study is its longitudinal design with a 14-year follow-up of a large cohort in which we evaluated circulating miRNA profiles and subsequent CRC risk using samples collected many years before diagnosis. Using the unique availability of detailed baseline information on environmental and genetic factors in the ESTHER study, we were able to simultaneously evaluate and compare the ability of three different types of risk scores for CRC risk prediction. However, there were some limitations to this study. Our findings await confirmation in independent prospective cohorts with long follow-up data. Thus, their exploratory nature has to be emphasized. Furthermore, we cannot exclude the possibility that extended time period of sample storage could have influenced our results. Nevertheless, pre-analytical and storage conditions were of high quality, and previous research suggests that prolonged storage has minimal effects on serum miRNA expression levels^50^. Finally, to what extent the miRNA deregulations identified in our study are CRC-specific needs to be explored.

In conclusion, our study demonstrates that while the contribution of ERS and PRS to CRC risk stratification is modest for the time being, miRNAs might serve as early indicators of CRC risk years prior to a diagnosis. We propose a miR-score observed to have altered expression in pre-diagnostic serum samples, which might be useful to identify high-risk populations for CRC screening. Our findings provide insight into how early circulating miRNA profiles indicative of CRC risk can be identified and suggest that this seems to be the case many years before CRC diagnosis. These findings could be most relevant for CRC screening. Future validation in extended prospective cohorts with large sample sizes and long follow-up data is needed to confirm the promise of miRNAs in CRC risk stratification. Finally, feasibility of implementing the proposed risk score in screening programs needs to be investigated.

## Methods

### Study design and populations

We adopted a two-step approach with a marker discovery and a marker validation phase. For the marker discovery phase, we used pre-treatment plasma samples from patients with newly diagnosed CRC and from controls without CRC recruited between 2016 and 2019 in the context of the GEKKO study. Briefly, the study includes two arms. In arm A, participants who underwent colonoscopy screening in medical practices and clinics in and around Heidelberg, Germany were recruited. In arm B, patients diagnosed with gastrointestinal, lung or breast cancer at the University Hospital Heidelberg were recruited. Participants filled out questionnaires (regarding socio-demographic characteristics, lifestyle factors) and provided biospecimens (blood, saliva, urine, stool, and breath condensate) which were processed in a central laboratory and stored in a biobank at −80 °C within 4 h. Colonoscopy reports (arm A) and hospital discharge letters (arm B) were provided by the treating physicians. The study was approved by the ethics committees of the Medical Faculties of the University Heidelberg (S-392/2015), the Eberhard Karls University, and the University Hospital Tübingen (876/2017BO2), the physicians’ boards of Baden-Württemberg (B-F-2016-034) and of Rhineland Palatinate (2018-13334_5). All participants provided written informed consent.

For the marker validation phase, we used serum samples from the ESTHER study, an ongoing population-based cohort study conducted in Saarland, Germany. Details of the ESTHER study design have been described previously^51^. In total, 9949 participants aged 50–75 years were recruited between July 2000 and December 2002 by their general practitioners in the context of a general health screening examination, and they have been regularly followed-up thereafter. Information on socio-demographic characteristics, lifestyle factors, and health status at baseline was obtained by standardized self-administered questionnaires. In addition, biological samples (blood, stool, and urine) were collected and stored at −80 °C until analysis. Prevalent and incident cancers were determined by record linkage with data from the Saarland Cancer Registry. The study was approved by the ethics committees of the Medical Faculty of the University of Heidelberg and of the state medical board of Saarland, Germany. All participants provided written informed consent.

In the marker discovery phase, we identified potential miRNA candidates using genome-wide profiling with NGS in plasma samples from a retrospective set (discovery set) of the GEKKO study that included 20 newly diagnosed CRC cases (from GEKKO arm B) and 20 controls free of colorectal neoplasm (from GEKKO arm A) matched by age and sex. Further candidates were identified through a literature review. MiRNA candidates obtained from the two sources were then measured in a case-cohort approach in baseline serum samples from incident CRC cases identified within 14 years of follow-up and randomly selected controls in the ESTHER study. More specifically, we used qPCR to profile miRNAs in serum collected at baseline from 198 participants with incident CRC and 178 randomly selected participants without diagnosis of CRC until the end of 2016 from participants enrolled during the first 6 months of recruitment (Fig. 1).

### MiRNA discovery by next-generation sequencing (NGS)

Plasma samples from the discovery set (GEKKO study) were thawed on ice and then centrifuged at 3000 × g for 5 min at 4 °C. RNA was isolated using an miRNeasy Plasma/Serum Kit (QIAGEN) as per the manufacturer’s instructions. Five μl total RNA was converted into miRNA NGS libraries using the QIAseq miRNA Library Kit (QIAGEN) as per the manufacturer’s instructions. Adapters containing unique molecular identifiers were ligated to the RNA before conversion to cDNA. After PCR (22 cycles), the samples were purified. Library preparation quality control was performed using either Bioanalyzer 2100 (Agilent, Santa Clara, California, United States) or TapeStation 4200 (Agilent). The libraries were pooled in equimolar ratios and sequenced on a NextSeq 500 sequencing instrument as per manufacturer’s instructions. FASTQ files were generated using the bcl2fastq software (version 2.2.0, Illumina Inc.) and checked using the FastQC tool. The reads were mapped to the GRCh37 reference genome using Bowtie2 (version 2.2.2). Reads were normalized using TMM method^52^. Differential expression analysis was performed using edgeR (version 3.12.1).

### Selecting miRNA candidates for validation

For the 912 miRNAs profiled using NGS, the raw data was normalized to TMM values and an exact test on the negative binomial distribution was applied to discover differentially expressed miRNAs between CRC cases and controls. The *P* values were adjusted to the number of comparisons using the Benjamini–Hochberg method, which yields a FDR to control Type I error^53^. 34 miRNAs were found to be differentially expressed between CRC patients and controls (Supplementary Table 1). Candidates were then selected using the following inclusion criteria: 1. FDR < 0.05; 2. TMM >10 in either case or control group and; 3. |log_2_FC| > 1. Twenty miRNAs meeting these criteria were selected as NGS candidates.

To select additional candidates from the literature, we searched PubMed for publications until 25^th^ April 2019 reporting plasma and serum miRNAs with externally or internally validated AUC values to discriminate CRC patients from controls. Search terms are provided in (Supplementary Note 1). We found 25 relevant publications which reported 64 unique miRNAs (as a single entity or as a panel) (Supplementary Table 2). Among the 64 literature candidates, we identified 8 miRNAs (miR-202-3p, miR-4669, miR-422a, miR-1290, miR-18b-5p, miR-17-3p, miR-31-5p and miR-204-5p) also detected by NGS, but not meeting the criterion for expression levels (TMM > 10 in at least one of the groups). We excluded these miRNAs, after which 56 literature candidates were further taken into consideration. Finally, we selected 21 miRNAs (individual or combined as a panel) with the highest AUCs and not overlapping with the NGS candidates as literature candidates. In total, 41 miRNA candidates were selected for qPCR profiling in the prospective set.

### MiRNA validation by quantitative real-time PCR (qPCR)

Serum samples from the prospective cohort (ESTHER study) were thawed on ice and centrifuged at 3000 × g for 5 min at 4 °C. Total RNA was extracted from the samples using miRCURY™ RNA Isolation Kit – Biofluids (QIAGEN, Germany) as per manufacturer’s instructions. Two μl RNA was reversely transcribed in ten μl reactions using the miRCURY LNA RT Kit (QIAGEN). cDNA was diluted 50x and assayed in ten ul PCR reactions according to the protocol for miRCURY LNA miRNA PCR. In a pre-analytical phase, spike-in controls UniSp2, UniSp4, and UniSp6 were added to control for RNA extraction efficiency and possible cDNA synthesis inhibitors. Hemolysis was assessed by determining the levels of miR‑451 and miR‑23a via qPCR. miR‑451 is expressed in red blood cells and miR‑23a is relatively stable in serum and not affected by hemolysis^54–56^. A Cq ratio between miR‑23a and miR‑451 higher than 7.0 was considered indicative of sample hemolysis^57^. Corresponding samples were excluded from further analysis.

For samples meeting the quality control criteria, each miRNA was assayed once on a custom panel using miRCURY LNA SYBR Green master mix. The primers for miRNAs are listed in Supplementary Table 4. Negative controls excluding template from the reverse transcription reaction were performed and profiled like the samples. The amplification was performed in 384 well plates on a LightCyclerG 480 Real-Time PCR System (Roche). The amplification curves were analyzed using the Roche LC software (version 1.5.0), both for the determination of Cq (Cq was calculated as the 2nd derivative) and for melting curve analysis. The amplification efficiency was calculated using algorithms similar to the LinReg software^58^. All assays were inspected for distinct melting curves and the melting temperature was checked to be within known specifications for the assay. Furthermore, assays within 5 Cq of the negative control or Cq>37 were excluded from further analysis. Detectable miRNAs were those with a Cq value <40.

All laboratory analyses were performed blinded with respect to disease status or findings at colonoscopy.

### Statistical analysis

#### qPCR data normalization and development of a microRNA risk score (miR-score)

Using the NormFinder^59^ method, a combination of three miRNAs (miR-93-5p, miR-1246 and miR-223-3p) exhibiting the highest stability across all samples (stability value = 1.04 × 10^−3^) was identified as normalizers. Samples with missing values for any of the normalizers were excluded from further analysis (*n* = 14). After selection of three normalizers, 38 miRNAs remained as candidates for evaluation in the validation phase. Of the 38 miRNAs, seven were detectable in at least 99% of the included samples and were identified as informative miRNAs. Samples with missing values for any of the informative miRNAs were excluded from further analysis (*n* = 9) and the remaining were included in the prospective set. The data were normalized to the average Cq value of the normalizers. The informative miRNAs were then utilized as a panel for fitting a logistic regression model on CRC risk, based on the prospective set. A miR-score was calculated for each participant by summing the observed expression levels of the seven miRNAs weighted by the estimated regression coefficients in the prospective set.

#### Environmental risk score (ERS)

For the prospective set, information on environmental risk factors, including sociodemographic and lifestyle factors was extracted from participants’ questionnaires administered at baseline. Considering the availability of variables, we applied a modified version of a previously derived ERS^36^ to predict the presence of CRC in our prospective set. The previously derived ERS used a variable ‘waist circumference’ which was not available in the ESTHER study and hence we replaced it with ‘body mass index’. Both variables are positively correlated and commonly used to assess weight-related health risks^60^. The ERS for each participant was built by summing up the score points for age (0 for <55 years, 1 for 55 to <60 years, 2 for 60 to <65 years, 3 for 65 to <70 years, or 4 for ≥70 years), sex (0 for female or 1 for male), first-degree relative with CRC (1 for ≥1 relative or 0 for other), body mass index (0 for < 25, 1 for 25 to <30, or 2 for ≥30 kg/m^2^), and cigarette smoking (0 for 0 pack-years, 2 for >0 to <30 pack-years, or 4 for ≥30 pack-years). Missing data for first-degree relative with CRC (*n* = 5, 1.3%), and cigarette smoking pack-years (*n* = 31, 8.1%) were imputed by chained equations^61^.

#### Polygenic risk score (PRS)

Extracted DNA from blood cell collected at baseline was genotyped using the Illumina OncoArray BeadChip (for 82% of the participants) and Global Screening Array (for 18% of the participants). Quality control of the genotype data was performed following a standardized protocol^62^. Missing genotypes (~40 million SNPs) were imputed using Haplotype Reference Consortium (version r1.1.2016) as reference panel within the Michigan Imputation Server. PLINK (version 1.9) was used to extract SNPs for the required region of interest. For building the PRS, a very recently reported set of 140 SNPs that were identified to be associated with a higher risk of CRC^37^ were considered. The PRS for each participant was calculated as a weighted sum of risk alleles using weights reported by Thomas et al.^37^.

#### Associations of the risk scores with CRC risk

To use the scores as risk stratification tools, the participants were stratified into five risk categories using quintile thresholds of the scores in the controls. Based on logistic regression models, ORs along with 95% CIs were estimated for CRC incidence taking the middle quintile as the reference group. Models were calculated first without adjusting for any confounders (Model 1); then additionally adjusting for age and sex (Model 2).

#### Risk prediction by individual and combined risk scores for CRC

In the prospective set, predictive performance of the miR-score was measured using AUC and Brier score. Potential over-optimism was accounted for by applying the .632+ bootstrapping method^63^ with 1000 replications. In addition to exploring the predictive ability of the miR-score over the entire period of follow-up, analyses were repeated with follow-up time restricted to the initial three years after recruitment and to subsequent years. Among participants with all scores available, predictive performance was evaluated for individual miR-score, ERS, and PRS as well as different combinations of the scores using AUCs and Brier scores. For the individual miR-score, as well as the score combinations including miR-score, .632+ bootstrap was applied to adjust for potential over-estimation of predictive performance.

#### Deregulation of individual informative miRNAs

For the prospective set, we performed Mann–Whitney test to compare expression levels of individual informative miRNAs between cases and controls. We adjusted the *P* values to the number of comparisons using the Bonferroni-Holm method^64^. The relative expression levels were calculated using 2^−ΔCt^ method^65^. The correlation of expression levels of individual informative miRNAs across participants of the prospective set was assessed by Spearman correlation coefficients.

All statistical analyses were performed with statistical software R (version 3.6.1) (R Core Team, 2016), together with R packages “mice” (version 3.12.0), “ModelGood” (version 1.0.9) and ‘pROC’ (version 1.16.2). For all tests, two-sided *P* values of 0.05 or less were considered to be statistically significant.

### Reporting summary

Further information on research design is available in the Nature Research Reporting Summary linked to this article.

## Supplementary information

Supplementary Information

Reporting Summary

## Data Availability

All miRNA sequencing data that support the findings of this study have been deposited in the European Genome-Phenome Archive (EGA) under restricted access with the accession code: EGAS00001005030. OncoArray and Global Screening Array genotype data have been deposited in the EGA under restricted access with the accession code: EGAS00001005411. The data are not publicly available due to them containing information that could compromise research participant privacy/consent. If you need to request access to this data, please contact: Petra Schrotz-King, email: petra.schrotz-king@nct-heidelberg.de. Data including miRNA qPCR data and relevant environmental risk factor data are available on reasonable request from the corresponding author (H.B.). All other relevant data are available within the article and its Supplementary Information file.
