# Supplementary material for: A microRNA panel compared to environmental and polygenic scores for colorectal cancer risk prediction
Source: Nat Commun. 2021 Aug 10;12:4811. doi: 10.1038/s41467-021-25067-8 (PMC8355103; doi:10.1038/s41467-021-25067-8)
Supplement: Supplementary file 1 — Supplementary Information [file 41467_2021_25067_MOESM1_ESM.pdf]

Supplementary Information

A microRNA panel compared to environmental and polygenic scores for colorectal cancer risk prediction

Raut et al.

Supplementary Table 1. MicroRNAs significantly differentially expressed in the plasma of colorectal cancer patients compared to controls in the discovery set.

	microRNA	TMM Cases	TMM Controls	log_2FC	*P* value[#]	Corrected *P* value[$]
1	**miR-370-3p**	90.43	229.24	-1.57	8.36E-06	5.97E-03
2	**miR-409-3p**	482.34	1211	-1.51	1.38E-05	5.97E-03
3	**miR-382-5p**	566.65	1432.08	-1.41	1.96E-05	5.97E-03
4	**miR-432-5p**	717.11	1572.68	-1.38	3.40E-05	7.75E-03
5	**miR-379-5p**	89.41	188.06	-1.58	4.77E-05	8.70E-03
6	**miR-134-5p**	226.92	599.91	-1.43	6.37E-05	9.68E-03
7	miR-16-5p	607674.17	305625.09	0.88	7.74E-05	1.01E-02
8	**miR-485-3p**	157.22	320.73	-1.31	9.35E-05	1.07E-02
9	**miR-32-5p**	183.64	74.28	1.04	1.86E-04	1.54E-02
10	**miR-323b-3p**	22.25	50.54	-1.21	1.96E-04	1.54E-02
11	**miR-766-5p**	4.29	10.68	-1.23	1.98E-04	1.54E-02
12	**let-7a-3p**	11.5	5.19	1.16	2.03E-04	1.54E-02
13	**miR-20b-5p**	563.54	220.09	1.09	2.39E-04	1.68E-02
14	**miR-337-5p**	8.52	18.15	-1.08	2.91E-04	1.73E-02
15	miR-20a-5p	813.66	476.42	0.72	2.99E-04	1.73E-02
16	miR-7-5p	622.78	326.57	0.82	3.04E-04	1.73E-02
17	**miR-664b-5p**	10.52	22.35	-1.18	3.54E-04	1.81E-02
18	**miR-431-5p**	68.57	137.01	-1.16	3.58E-04	1.81E-02
19	miR-19a-3p	285.23	141.48	0.82	4.16E-04	1.99E-02
20	miR-576-5p	72.75	36.86	0.88	5.49E-04	2.50E-02
21	miR-6750-5p	4.49	1.24	1.55	6.62E-04	2.78E-02
22	**miR-144-5p**	334.6	133.78	1.09	6.72E-04	2.78E-02
23	miR-106b-3p	530.78	289.81	0.72	7.32E-04	2.90E-02
24	**miR-3613-5p**	152.88	69.83	1.06	7.90E-04	3.00E-02
25	miR-101-3p	7452.74	3389.57	0.94	8.75E-04	3.19E-02
26	miR-3614-5p	2.58	0.62	1.83	1.03E-03	3.63E-02
27	**miR-654-3p**	61.48	114.47	-1.07	1.14E-03	3.70E-02
28	**miR-148b-5p**	18.95	9.87	1.01	1.14E-03	3.70E-02
29	miR-363-3p	261.71	126.53	0.86	1.18E-03	3.70E-02
30	miR-182-5p	1222.67	590.83	0.90	1.23E-03	3.73E-02
31	miR-486-3p	135.46	69.59	0.90	1.29E-03	3.80E-02
32	**miR-485-5p**	24.97	56.57	-1.23	1.44E-03	4.12E-02
33	miR-329-3p	3.09	7.46	-1.23	1.61E-03	4.44E-02
34	miR-30e-5p	6820.84	4594.36	0.51	1.73E-03	4.65E-02

[#] two-sided *P* value estimated by an exact test on the negative binomial distribution.
[$] Multiple testing correction by the method of Benjamini and Hochberg[1]

Note: MicroRNAs in bold were selected for further validation by quantitative real-time polymerase chain reaction.

Abbreviations: TMM, average trimmed mean of M (log expression ratio)-values/; FC, fold change between cases and controls.

Supplementary Table 2. Sixty-four unique plasma or serum microRNAs for colorectal cancer detection identified by literature search and sorted by their performance.

	microRNA	Selected as single/ panel microRNA	AUC (95% CI) from validation rounds [References]	Other citations for individual microRNA
1	**miR-1246**	Panel	0.96 (0.94- 0.98)[2]	
2	miR-202-3p[a]			
3	**miR-21-3p**			
4	**miR-1229-3p**			
5	**miR-532-3p**			[3]
6	miR-31-5p[a]	Panel	0.96 (0.87- 0.99)[4]	[5,6]
7	**miR-141-3p**			[6]
8	**miR-224-3p**			
9	**miR-576-5p**			
10	miR-4669[a]			
11	**miR-19a-3p**	Panel	0.95 (0.91- 0.98)[7]	[8,9]
12	**miR-223-3p**			[6]
13	**miR-92a-3p**			[5,6,10,11]
14	miR-422a[a]			
15	**miR-21-5p**	Single	0.93 (0.89- 0.96)[12]	[5,9,13-16]
16	**let-7g-5p**	Panel	0.92 (0.87- 0.98)[5]	
17	**miR-181b-5p**			
18	**miR-203a-3p**			
19	**miR-23a-3p**	Panel	0.92[17]	
20	**miR-142-5p**			
21	**miR-27a-3p**			
22	**miR-376c-3p**			
23	miR-1290[a]	Panel	0.91 (0.85- 0.96)[18]	[19]
24	miR-320d			
25	miR-409-3p[#]	Panel	0.90 (0.75- 0.95)[20]	
26	**miR-7-5p**			
27	**miR-93-5p**			
28	miR-17-5p	Panel	0.90 (0.81- 0.98)[21]	[15]
29	miR-18a-5p			[6]
30	miR-103a-3p			
31	miR-181a-5p			
32	miR-127-3p			
33	miR-18b-5p[a]			
34	miR-151a-5p			
35	**miR-182-5p**	Single	0.89 (0.82- 0.96)[22]	
36	miR-29a-3p	Panel with miR-92a-3p	0.88 (0.83- 0.94)[11]	[3]
37	miR-375-3p	Panel	0.85 (0.78- 0.92)[23]	
38	miR-206			

	microRNA	Selected as single/ panel microRNA	AUC (95% CI) from validation rounds [References]	Other citations for individual microRNA
39	miR-19b-3p	Panel with miR-19a-3p	0.84 (0.76- 0.92)[8]	
40	miR-15b-5p			[15]
41	miR-20a-5p	Panel with miR-182-5p	0.83 (0.75- 0.91)[22]	
42	miR-431-5p[#]	Panel	0.83 (0.73- 0.93)[13]	
43	miR-139-3p			
44	miR-425-5p	Panel with miR-19a-3p & miR-21-5p	0.83 (0.71- 0.95)[9]	
45	miR-378	Single	0.80 (0.68- 0.91)[14]	
46	miR-221-3p	Panel	0.79[6]	
47	miR-191-5p			
48	miR-24-3p			
49	miR-760	Single	0.79 (0.71- 0.86)[24]	
50	miR-601	Panel with miR-760	0.79 (0.72- 0.87)[24]	
51	miR-96-5p	Single	0.74 (0.65- 0.83)[25]	
52	miR-17-3p[a]	Single	0.72 (0.63- 0.80)[10]	
53	miR-26b-5p	Single	0.71 (0.63- 0.79)[15]	
54	miR-142-3p	Single	0.71[26]	
55	miR-145-5p	Single	0.67[15]	
56	miR-26a-5p	Single	0.67[26]	
57	miR-10b-5p	Single	0.65 (0.58- 0.71)[6]	
58	miR-25-3p	Single	0.64 (0.58- 0.71)[6]	
59	miR-133a-3p	Single	0.63[3]	
60	miR-497-5p	Single	0.58 (0.51- 0.64)[6]	
61	miR-106b-5p	Single	0.57[3]	
62	miR-204-5p[a]	Single	0.49 (0.34- 0.65)[14]	
63	miR-135b-5p	Single	0.48 (0.32- 0.64)[14]	
64	miR-342-3p	Single	0.44[3]	

[a] excluded as a potential literature miRNA candidate since it did not meet the criterion for expression level (TMM > 10 in at least one of the groups) in the next-generation sequencing (NGS) analysis
[#] already included as an NGS miRNA candidate
Note: miRNAs in bold were selected for quantitative real-time polymerase chain reaction profiling in the prospective set.

Abbreviations: miRNA, microRNA; AUC, area under the receiver-operating-characteristic curve; CI, confidence interval.

Supplementary Table 3. Colorectal cancer risk prediction by microRNA risk score in the prospective set ($N_{case/control}$ = 198/178) with respect to time to diagnosis in cases.

Population	$N_{case/control}$	AUC (95% CI)	Brier score
CRC cases diagnosed in ≤ 1yr vs. Controls	15/178	0.891 (0.803-0.978)	0.167
CRC cases diagnosed in ≤ 2yrs vs. Controls	31/178	0.796 (0.696-0.895)	0.173
CRC cases diagnosed in ≤ 3yrs vs. Controls	45/178	0.780 (0.693-0.867)	0.176
CRC cases diagnosed after 3yrs vs. Controls	153/178	0.816 (0.770-0.862)	0.172

Abbreviations: CRC, colorectal cancer; AUC, area under the receiver-operating-characteristic curve; CI, confidence interval.

Supplementary Table 4. Information on the quantitative real-time polymerase chain reaction primers (QIAGEN).

	microRNA	Accession	Catalog number	Sequence
1	miR-370-3p	MIMAT0000722	YP00204011	GCCUGCUGGGGUGGAACCUGGU
2	miR-409-3p	MIMAT0001639	YP00204358	GAAUGUUGCUCGGUGAACCCCU
3	miR-382-5p	MIMAT0000737	YP00204169	GAAGUUGUUCGUGGUGGAUUCG
4	miR-432-5p	MIMAT0002814	YP00204776	UCUUGGAGUAGGUCAUUGGGUGG
5	miR-379-5p	MIMAT0000733	YP00205658	UGGUAGACUAUGGAACGUAGG
6	miR-134-5p	MIMAT0000447	YP00205989	UGUGACUGGUUGACCAGAGGGG
7	miR-485-3p	MIMAT0002176	YP00206055	GUCAUACACGGCUCUCCUCUCU
8	miR-485-5p	MIMAT0002175	YP02112548	AGAGGCUGGCCGUGAUGAAUUC
9	miR-32-5p	MIMAT0000090	YP00204792	UAUUGCACAUUACUAAGUUGCA
10	miR-323b-3p	MIMAT0015050	YP02100923	CCCAAUACACGGUCGACCUCUU
11	miR-766-5p	MIMAT0022714	YP02113546	AGGAGGAAUUGGUGCUGGUCUU
12	let-7a-3p	MIMAT0004481	YP00206084	CUAUACAAUCUACUGUCUUUC
13	miR-20b-5p	MIMAT0001413	YP00204755	CAAAGUGCUCAUAGUGCAGGUAG
14	miR-337-5p	MIMAT0004695	YP00204391	GAACGGCUUCAUACAGGAGUU
15	miR-664b-5p	MIMAT0022271	YP02109261	UGGGCUAAGGGAGAUGAUUGGGUA
16	miR-431-5p	MIMAT0001625	YP00204737	UGUCUUGCAGGCCGUCAUGCA
17	miR-144-5p	MIMAT0004600	YP00204670	GGAUAUCAUCAUAUACUGUAAG
18	miR-3613-5p	MIMAT0017990	YP02119046	UGUUGUACUUUUUUUUUUGUUC
19	miR-654-3p	MIMAT0004814	YP00204054	UAUGUCUGCUGACCAUCACCUU
20	miR-148b-5p	MIMAT0004699	YP00204735	AAGUUCUGUUAUACACUCAGGC
21	miR-1246	MIMAT0005898	YP00205630	AAUGGAUUUUUGGAGCAGG
22	miR-21-3p	MIMAT0004494	YP00204302	CAACACCAGUCGAUGGGCUGU
23	miR-1229-3p	MIMAT0005584	YP00206036	CUCUCACCACUGCCCUCCCACAG
24	miR-532-3p	MIMAT0004780	YP00204003	CCUCCCACACCCAAGGCUUGCA
25	miR-141-3p	MIMAT0000432	YP00204504	UAACACUGUCUGGUAAAGAUGG
26	miR-224-3p	MIMAT0009198	YP00204629	AAAAUGGUGCCCUAGUGACUACA
27	miR-576-5p	MIMAT0003241	YP00206064	AUUCUAAUUUCUCCACGUCUUU
28	miR-19a-3p	MIMAT0000073	YP00205862	UGUGCAAAUCUAUGCAAAACUGA
29	miR-223-3p	MIMAT0000280	YP00205986	UGUCAGUUUGUCAAAUACCCCA
30	miR-92a-3p	MIMAT0000092	YP00204258	UAUUGCACUUGUCCCGGCCUGU
31	miR-21-5p	MIMAT0000076	YP00204230	UAGCUUAUCAGACUGAUGUUGA
32	let-7g-5p	MIMAT0000414	YP00204565	UGAGGUAGUAGUUUGUACAGUU
33	miR-181b-5p	MIMAT0000257	YP00204530	AACAUUCAUUGCUGUCGGUGGGU
34	miR-203a-3p	MIMAT0000264	YP00205914	GUGAAAUGUUUAGGACCACUAG
35	miR-23a-3p	MIMAT0000078	YP00204772	AUCACAUUGCCAGGGAUUUCC
36	miR-142-5p	MIMAT0000433	YP00204722	CAUAAAGUAGAAAGCACUACU
37	miR-27a-3p	MIMAT0000084	YP00206038	UUCACAGUGGCUAAGUUCCGC
38	miR-376c-3p	MIMAT0000720	YP00204442	AACAUAGAGGAAAUUCCACGU
39	miR-7-5p	MIMAT0000252	YP00205877	UGGAAGACUAGUGAUUUUGUUGU
40	miR-93-5p	MIMAT0000093	YP00204715	CAAAGUGCUGUUCGUGCAGGUAG
41	miR-182-5p	MIMAT0000259	YP00206070	UUUGGCAAUGGUAGAACUCACACU

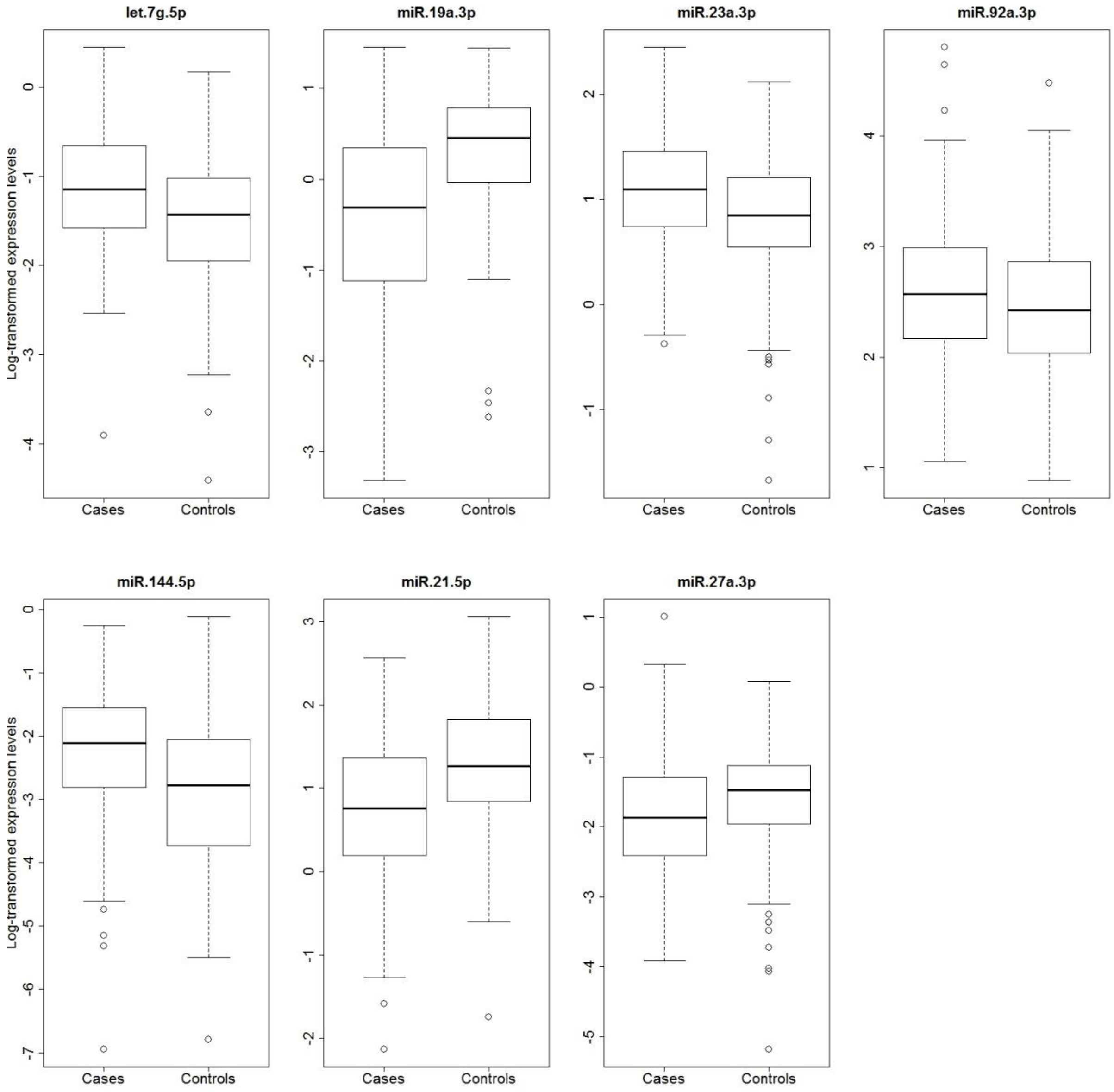

Supplementary Fig. 1. Box-plots showing serum miRNA expression levels in the prospective set ($N_{case/control}$ = 198/178) determined by quantitative real-time polymerase chain reaction. Expression levels of miRNAs are normalized to the average Cq value of miR-93-5p, miR-1246 and miR-223-3p and are represented as log-transformed (-ΔCq) values. The lines inside the boxes denote the medians. The boxes mark the interval between the 25th and 75th percentiles. The whiskers extend to the observations which are no more than 1.5 times the length of the box (interquartile range) away from the box. More extreme observations are considered outliers and are indicated as single circle symbols.

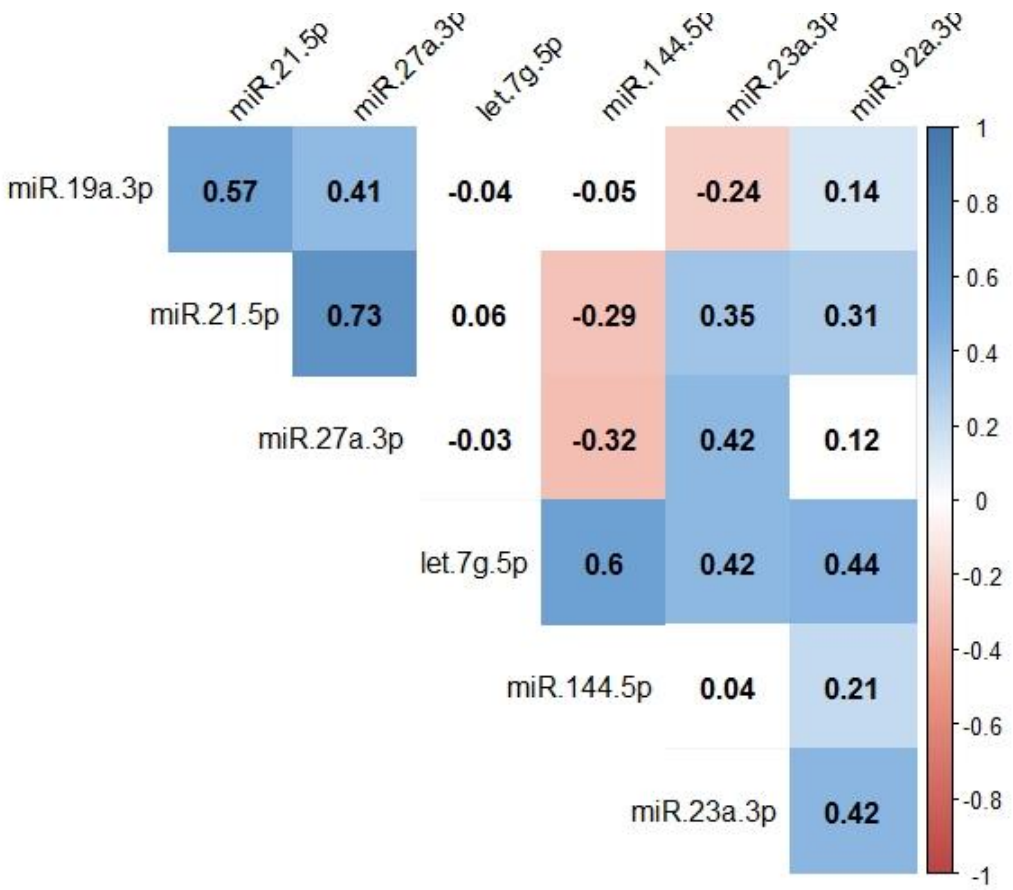

Supplementary Fig. 2. Spearman's correlation coefficients of microRNA expression levels in the prospective set ($N_{case/control}$ = 198/178) (*p* <0.05 if not blank).

Supplementary Note 1. PubMed search terms.

[(colorectal OR colon OR colonic OR rectal OR rectum)) AND (cancer OR carcinoma OR adenoma OR neoplasm OR tumor OR malignancy) AND (plasma OR serum OR sera) AND (microRNA OR micro-RNA OR "microRNA" OR miRNA OR mi-RNA OR miR OR let-7) AND (predict OR detect OR diagnose OR diagnosis OR screen OR marker OR biomarker) AND ("receiver operating characteristic" OR ROC OR AUC) AND (valid*)].

Supplementary References

1 Benjamini, Y. & Hochberg, Y. Controlling the false discovery rate: a practical and powerful approach to multiple testing. *J. R. Stat. Soc. Ser. B (Methodol.)* **57**, 289-300 (1995).

2 Guo, S. *et al.* A 5-serum miRNA panel for the early detection of colorectal cancer. *Onco Targets Ther.* **11**, 2603-2614 (2018).

3 Luo, X., Stock, C., Burwinkel, B. & Brenner, H. Identification and evaluation of plasma microRNAs for early detection of colorectal cancer. *PLoS One* **8**, e62880 (2013).

4 Wang, Y. N., Chen, Z. H. & Chen, W. C. Novel circulating microRNAs expression profile in colon cancer: a pilot study. *Eur. J. Med. Res.* **22**, 51 (2017).

5 Wang, J. *et al.* Identification of a circulating microRNA signature for colorectal cancer detection. *PLoS One* **9**, e87451 (2014).

6 Chang, P. Y. *et al.* MicroRNA-223 and microRNA-92a in stool and plasma samples act as complementary biomarkers to increase colorectal cancer detection. *Oncotarget* **7**, 10663-10675 (2016).

7 Zheng, G. *et al.* Serum microRNA panel as biomarkers for early diagnosis of colorectal adenocarcinoma. *Br. J. Cancer* **111**, 1985-1992 (2014).

8 Giraldez, M. D. *et al.* Circulating microRNAs as biomarkers of colorectal cancer: results from a genome-wide profiling and validation study. *Clin. Gastroenterol. Hepatol.* **11**, 681-688 (2013).

9 Zhu, M. *et al.* A panel of microRNA signature in serum for colorectal cancer diagnosis. *Oncotarget* **8**, 17081-17091 (2017).

10 Ng, E. K. *et al.* Differential expression of microRNAs in plasma of patients with colorectal cancer: a potential marker for colorectal cancer screening. *Gut* **58**, 1375-1381 (2009).

11 Huang, Z. *et al.* Plasma microRNAs are promising novel biomarkers for early detection of colorectal cancer. *Int. J. Cancer* **127**, 118-126 (2010).

12 Toiyama, Y. *et al.* Serum miR-21 as a diagnostic and prognostic biomarker in colorectal cancer. *J. Natl. Cancer Inst.* **105**, 849-859 (2013).

13 Kanaan, Z. *et al.* Plasma miR-21: a potential diagnostic marker of colorectal cancer. *Ann. Surg.* **256**, 544-551 (2012).

14 Zanutto, S. *et al.* Circulating miR-378 in plasma: a reliable, haemolysis-independent biomarker for colorectal cancer. *Br. J. Cancer* **110**, 1001-1007 (2014).

15 Pan, C. *et al.* Systematic literature review and clinical validation of circulating microRNAs as diagnostic biomarkers for colorectal cancer. *Oncotarget* **8**, 68317-68328 (2017).

16 Wikberg, M. L., Myte, R., Palmqvist, R., van Guelpen, B. & Ljuslinder, I. Plasma miRNA can detect colorectal cancer, but how early? *Cancer Med.* **7**, 1697-1705 (2018).

17 Vychytilova-Faltejskova, P. *et al.* Serum-based microRNA signatures in early diagnosis and prognosis prediction of colon cancer. *Carcinogenesis* **37**, 941-950 (2016).

18 Liu, X. *et al.* Circulating miR-1290 and miR-320d as Novel Diagnostic Biomarkers of Human Colorectal Cancer. *J. Cancer* **10**, 43-50 (2019).

19 Imaoka, H. *et al.* Circulating microRNA-1290 as a novel diagnostic and prognostic biomarker in human colorectal cancer. *Ann. Oncol.* **27**, 1879-1886 (2016).

20 Wang, S. *et al.* A plasma microRNA panel for early detection of colorectal cancer. *Int. J. Cancer* **136**, 152-161 (2015).
21 Zhang, H. *et al.* A panel of seven-miRNA signature in plasma as potential biomarker for colorectal cancer diagnosis. *Gene* **687**, 246-254 (2018).
22 Liu, X. *et al.* Elevated circulating miR-182 acts as a diagnostic biomarker for early colorectal cancer. *Cancer Manag. Res.* **10**, 857-865 (2018).
23 Xu, L. *et al.* The expression of microRNA-375 in plasma and tissue is matched in human colorectal cancer. *BMC Cancer* **14**, 714 (2014).
24 Wang, Q. *et al.* Plasma miR-601 and miR-760 are novel biomarkers for the early detection of colorectal cancer. *PLoS One* **7**, e44398 (2012).
25 Sun, Y. *et al.* Examining plasma microRNA markers for colorectal cancer at different stages. *Oncotarget* **7**, 11434-11449 (2016).
26 Ghanbari, R. *et al.* Downregulation of Plasma MiR-142-3p and MiR-26a-5p in Patients With Colorectal Carcinoma. *Iranian journal of cancer prevention* **8**, e2329 (2015).
