# Supplementary material for: A microRNA panel compared to environmental and polygenic scores for colorectal cancer risk prediction
Source: Nat Commun. 2021 Aug 10;12:4811. doi: 10.1038/s41467-021-25067-8 (PMC8355103; doi:10.1038/s41467-021-25067-8)
Supplement: Supplementary file 2 — Reporting Summary [file 41467_2021_25067_MOESM2_ESM.pdf]

nature research

Corresponding author(s): Prof. Hermann Brenner

Last updated by author(s): May 10, 2021

Reporting Summary

Nature Research wishes to improve the reproducibility of the work that we publish. This form provides structure for consistency and transparency in reporting. For further information on Nature Research policies, see our Editorial Policies and the Editorial Policy Checklist.

Statistics

For all statistical analyses, confirm that the following items are present in the figure legend, table legend, main text, or Methods section.

n/a	Confirmed	
☐	☒	The exact sample size (*n*) for each experimental group/condition, given as a discrete number and unit of measurement
☐	☒	A statement on whether measurements were taken from distinct samples or whether the same sample was measured repeatedly
☐	☒	The statistical test(s) used AND whether they are one- or two-sided<br>*Only common tests should be described solely by name; describe more complex techniques in the Methods section.*
☐	☒	A description of all covariates tested
☐	☒	A description of any assumptions or corrections, such as tests of normality and adjustment for multiple comparisons
☐	☒	A full description of the statistical parameters including central tendency (e.g. means) or other basic estimates (e.g. regression coefficient) AND variation (e.g. standard deviation) or associated estimates of uncertainty (e.g. confidence intervals)
☐	☒	For null hypothesis testing, the test statistic (e.g. *F*, *t*, *r*) with confidence intervals, effect sizes, degrees of freedom and *P* value noted<br>*Give P values as exact values whenever suitable.*
☒	☐	For Bayesian analysis, information on the choice of priors and Markov chain Monte Carlo settings
☒	☐	For hierarchical and complex designs, identification of the appropriate level for tests and full reporting of outcomes
☒	☐	Estimates of effect sizes (e.g. Cohen's *d*, Pearson's *r*), indicating how they were calculated

Our web collection on statistics for biologists contains articles on many of the points above.

Software and code

Policy information about availability of computer code

Data collection	Sequencing data FASTQ files were generated using the bcl2fastq software (version 2.2.0, Illumina Inc.) and checked using the FastQC tool. The reads were mapped to the GRCh37 reference genome using Bowtie2 (version 2.2.2).
Data analysis	Sequencing data differential expression analysis was performed using edgeR (version 3.12.1).<br>Missing genotypes (~40 million SNPs) were imputed using Haplotype Reference Consortium (version r1.1.2016) as reference panel within the Michigan Imputation Server. PLINK (version 1.9) was used to extract SNPs for the required region of interest.<br>The quantitative real-time polymerase chain reaction (qPCR) amplification curves were analyzed using the Roche LC software (version 1.5.0), both for the determination of raw quantification cycle (Cq) and for melting curve analysis.<br>Other statistical analyses, including imputation by chained equations, logistic regression and risk prediction analyses, were performed using R (version 3.6.1) (R Core Team, 2016) together with packages , 'mice' (version 3.12.0), ‘ModelGood’ (version 1.0.9) and 'pROC' (version 1.16.2).

For manuscripts utilizing custom algorithms or software that are central to the research but not yet described in published literature, software must be made available to editors and reviewers. We strongly encourage code deposition in a community repository (e.g. GitHub). See the Nature Research guidelines for submitting code & software for further information.

Data

Policy information about availability of data

All manuscripts must include a data availability statement. This statement should provide the following information, where applicable:

- Accession codes, unique identifiers, or web links for publicly available datasets
- A list of figures that have associated raw data
- A description of any restrictions on data availability

All microRNA (miRNA) sequencing data that support the findings of this study have been deposited in the European Genome-Phenome Archive (EGA) under restricted access with the accession code: EGAS00001005030. The data are not publicly available due to restrictions of informed consent. All other relevant data are available within Supplementary information, or are available on reasonable request from the corresponding author (H.B.).

Field-specific reporting

Please select the one below that is the best fit for your research. If you are not sure, read the appropriate sections before making your selection.

- [x] Life sciences
- [ ] Behavioural & social sciences
- [ ] Ecological, evolutionary & environmental sciences

For a reference copy of the document with all sections, see nature.com/documents/nr-reporting-summary-flat.pdf

Life sciences study design

All studies must disclose on these points even when the disclosure is negative.

Sample size	No sample-size calculation was performed. For both the discovery and prospective sets, we used all available samples from colorectal cancer (CRC) cases. To maintain a 50% cases and 50% controls ratio, we used an almost equal number of samples from controls.
Data exclusions	We excluded hemolytic samples and for the development of a miRNA risk score, we excluded samples in which any of the normalizer/informative (Cq values <40 in at least 99% of the samples) miRNAs was not expressed.
Replication	Twenty miRNA candidates selected from analysis of the NGS experiment were evaluated by qPCR experiment in an independent cohort of cases and controls. Of the twenty candidates, one was identified as an informative miRNA for which the findings were replicated.
Randomization	Not applicable since the samples are derived from fixed experimental groups.
Blinding	All laboratory analyses were performed blinded with respect to disease status or findings at colonoscopy.

Reporting for specific materials, systems and methods

We require information from authors about some types of materials, experimental systems and methods used in many studies. Here, indicate whether each material, system or method listed is relevant to your study. If you are not sure if a list item applies to your research, read the appropriate section before selecting a response.

Materials & experimental systems

n/a	Involved in the study	
x		Antibodies
x		Eukaryotic cell lines
x		Palaeontology and archaeology
x		Animals and other organisms
	x	Human research participants
x		Clinical data
x		Dual use research of concern

Methods

n/a	Involved in the study	
x		ChIP-seq
x		Flow cytometry
x		MRI-based neuroimaging

Human research participants

Policy information about studies involving human research participants

Population characteristics	The discovery set included 20 newly diagnosed CRC cases (from GEKKO arm B) and 20 controls free of colorectal neoplasm (from GEKKO arm A) matched by age and sex. Of the 19 cases with information about tumor stage at diagnosis, one was classified as stage 0, one as stage I, nine as stage II, four as stage III, and four as stage IV.<br>The prospective set included 198 participants with incident CRC and 178 randomly selected participants without diagnosis of CRC identified within 14 years of follow-up in the ESTHER study. Of the 153 cases with information about tumor stage at diagnosis, 14 were classified as stage 0, 20 as stage I, 61 as stage II, 30 as stage III, and 28 as stage IV.<br>The distribution of characteristics was largely similar across both, the discovery and prospective sets with the mean age at sampling being around 65 years and males representing >50% of population in both sets.
Recruitment	The discovery set participants were recruited between 2016-2019 in the context of the GEKKO study. Briefly, the study includes two arms. In arm A, participants who underwent colonoscopy screening in medical practices and clinics in and around Heidelberg, Germany were recruited. In arm B, patients diagnosed with gastrointestinal, lung or breast cancer at the University Hospital Heidelberg were recruited. Participants 30 years or older, speaking and understanding the German language, with no previous colonoscopy in the last 5 years, no personal history of CRC, and no inflammatory bowel disease were eligible to participate. The discovery set cases were from arm B and controls were from arm A. By using pre-treatment samples from patients with newly diagnosed CRC, we tried to avoid biases due to later stages of disease development and treatments. We tried to account for selection bias by matching cases and controls on the risk factors age and sex.<br><br>The prospective set participants were drawn from the ESTHER study, an ongoing statewide population based cohort study among older adults conducted in Saarland, Germany. Briefly, 9,949 male and female residents of Saarland aged 50-75 years with sufficient knowledge of the German language were recruited in 2000-2002 by their general practitioners during a routine health check-up aiming at early detection of cardiovascular diseases and diabetes. The study population has been shown to closely resemble the study population of a representative German national health survey within the corresponding age range carried out in 1998 with respect to major sociodemographic and health related characteristics. For our study, we used all available samples collected at baseline from incident CRC cases identified within 14 years of follow-up. We used samples from randomly selected controls (participants without diagnosis of CRC until the end of 2016) enrolled during the first 6 months of recruitment. To the best of our knowledge there were no sources of (self-)selection bias.
Ethics oversight	The GEKKO study was approved by the ethics committees of the Medical Faculties of the University Heidelberg (S-392/2015), the Eberhard Karls University and the University Hospital Tübingen (876/2017BO2), the physicians' boards of Baden-Württemberg (B-F-2016-034) and of Rhineland Palatinate (2018-13334_5). The ESTHER study was approved by the ethics committees of the Medical Faculty of the University of Heidelberg and of the state medical board of Saarland, Germany.

Note that full information on the approval of the study protocol must also be provided in the manuscript.
